# Silicon-based fluorescent platforms for copper(ii) detection in water†

**DOI:** 10.1039/d1ra02695j

**Published:** 2021-04-26

**Authors:** Mariangela Oggianu, Cristiana Figus, Suchithra Ashoka-Sahadevan, Noemi Monni, Daniela Marongiu, Michele Saba, Andrea Mura, Giovanni Bongiovanni, Claudia Caltagirone, Vito Lippolis, Carla Cannas, Enzo Cadoni, Maria Laura Mercuri, Francesco Quochi

**Affiliations:** Dipartimento di Scienze Chimiche e Geologiche, Università degli Studi di Cagliari, Complesso Universitario di Monserrato I-09042 Monserrato (CA) Italy mercuri@unica.it; Dipartimento di Fisica, Università degli Studi di Cagliari, Complesso Universitario di Monserrato I-09042 Monserrato (CA) Italy quochi@unica.it; INSTM, Cagliari Unit Via Giuseppe Giusti, 9 I-50121 Firenze Italy

## Abstract

The potential of silicon-based fluorescent platforms for the detection of trace toxic metal ions was investigated in an aqueous environment. To this aim, silicon chips were first functionalized with amino groups, and fluorescein organic dyes, used as sensing molecules, were then covalently linked to the surface *via* formation of thiourea groups. The obtained hybrid heterostructures exhibited high sensitivity and selectivity towards copper(ii), a limit of detection compatible with the recommended upper limits for copper in drinking water, and good reversibility using a standard metal–chelating agent. The fluorophore–analyte interaction mechanism at the basis of the reported fluorescence quenching, as well as the potential of performance improvement, were also studied. The herein presented sensing architecture allows, in principle, tailoring of the selectivity towards other metal ions by proper fluorophore selection, and provides a favorable outlook for integration of fluorescent chemosensors with silicon photonics technology.

## Abstract 2

Covalent linkage of fluorescein to silanized silicon chips yields solid-state platforms for detection of copper(ii) in water. This architecture represents a step forward towards the fabrication of sensors for remote water analysis applications.

## Graphical abstract

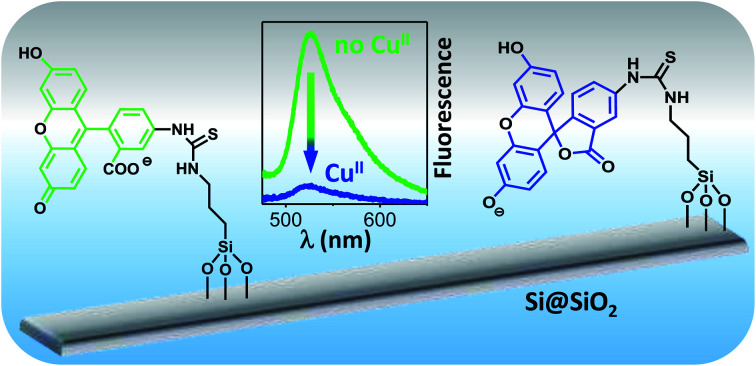

## Introduction

Several techniques are currently employed for detecting toxic metal ions at low concentrations in aqueous and biological environments, including Inductively Coupled Plasma Atomic Emission Spectrometry (ICP-AES),^1^ Inductively Coupled Plasma Mass Spectrometry (ICP-MS)^2^ and Atomic Absorption Spectrometry (AAS).^3^ These techniques provide excellent detection limits but involve high-cost instrumentation, time-consuming sample preparation and highly trained personnel. Colorimetric and fluorescence sensor devices are fast-growing technologies showing remarkable advantages over conventional techniques, such as fast response times, non-destructive analysis and remote operation, while attaining competitive performances in terms of detection limits, sensitivity, selectivity and reversibility by rational device design.^4–8^

Fluorescent probes, which have found widespread use in biomedical applications,^9,10^ are being increasingly studied for real-time and remote environmental monitoring such as detection of toxic metals in water and biological media.^11–15^ In a molecular-type approach, a fluorescent probe, which represents the sensitive part of the fluorescent device, basically consists of a recognition (binding) moiety linked to or included into a fluorescent species (usually an organic dye) whose emission properties, such as peak wavelength and quantum yield, are modified by the interaction with the analyte. The chemical nature of both the binding and fluorescent units can be tailored to optimize the probe for any specific analyte and/or application.

Most fluorescent probes are designed to work in solution. However, depending on the application, an improvement in the sensing performance can be obtained by covalently anchoring fluorescent probes to different substrates, including biomolecules,^16,17^ silica-based nanocomposites^18–21^ and metal–organic frameworks.^22–24^ Grafting fluorescent probes to silicon-based planar platforms represents a promising route towards sensor integration with silicon photonics technology and realization of Lab-on-Chip devices.^25,26^ To this aim, the sol–gel technique based on organosilane derivatives^27,28^ is one of the most suitable strategies for fabricating robust, low-cost and functional silicon-based platforms. In particular, the use of 3-aminopropyltriethoxysilane (APTES) as organosilane derivate, allows to insert amino groups capable to covalently link fluorophores on silicon surfaces, *via* the formation of amides, ureas, thioureas and imines as linking functional groups.^29–32^ Although step-by-step silanization of silicon substrates has already been investigated for the fabrication of fluorescent platforms,^33–35^ the potential of these platforms for the detection of trace metal ions in aqueous environment is yet to be demonstrated.

A fundamental study on silicon-based fluorescent platforms for detection of trace metal ions in aqueous environment is herein presented. Fluorescein isothiocyanate (FITC) was covalently linked to APTES-prefunctionalized silicon substrates *via* thiourea formation to realize silicon-based on-chip devices integrating both recognition and fluorescent units. On the one hand, FITC is highly suitable to serve as prototypical fluorescent sensing unit because of its remarkable photophysical properties;^36–38^ on the other hand, thiourea groups have been demonstrated effective in binding metal cations such as Cu^II^, Cd^II^, and Hg^II^ in water, in fact enabling metal-ion fluorescent sensors with competitive performances.^22,39–43^ Selectivity to copper(ii), high sensitivity and low limit of detection were reported. Successful surface regeneration was achieved using ethylenediaminetetraacetic acid (EDTA) chelating agent, yielding insight into the mechanism responsible for metal binding.

Among heavy metal ions, copper plays a crucial role in different biological processes,^44^ being essential for the functioning of many enzymes such as C oxidase (COX) and dopamine β-hydroxylase.^45^ Its accumulation in the human body can be harmful and even lead to genetic disorders such as Wilson's disease, and neurodegenerative disorders including Alzheimer's and Parkinson's disease.^46–48^ Consequently, EPA (US Environmental Protection Agency) and WHO (World Health Organization) have set the maximum copper level in drinking waters to 1.3 and 2 mg L^−1^, respectively. Therefore, the search for innovative metal-ion sensing devices represents still a challenge in the environmental field.

## Results and discussion

**Fig. 1:**
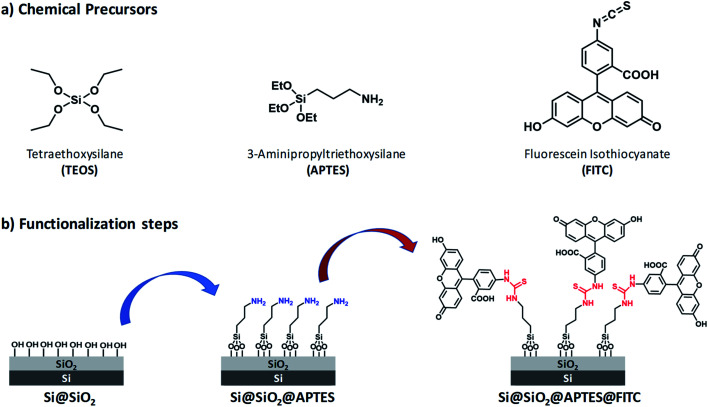
(a) Chemical precursors used for functionalization of (100)-oriented silicon chips. (b) Step 1: surface silica layer deposition bearing hydroxy groups *via* the sol–gel method using TEOS (left); Step 2: amino-functionalization of the silica surface with APTES (centre, amino groups highlighted in blue). Step 3: functionalization with FITC (right), leading to the formation of thiourea groups (highlighted in red).

Reagents and silicon surface modification steps are illustrated in Fig. 1. (100)-Oriented silicon chips were first coated with ultrathin silica layers by (i) the sol–gel technique by using tetraethoxysilane (TEOS) as silica precursor (Scheme S1, ESI†) and (ii) the spin-coating deposition method (Step 1). Subsequently, amino groups were introduced on the Si@SiO_2_ surface by using APTES (Step 2). Finally, FITC was covalently linked to the Si@SiO_2_@APTES surface *via* thiourea formation (Step 3).

Freshly prepared samples were investigated following a multivariate approach, namely, Contact-Angle (CA) measurements, Atomic-Force Microscopy (AFM), Reflectance and Fluorescence Spectroscopy. This way, it was possible to monitor how each functionalization step modified the morphological and optical properties of the silicon surface.^49^

**Fig. 2:**
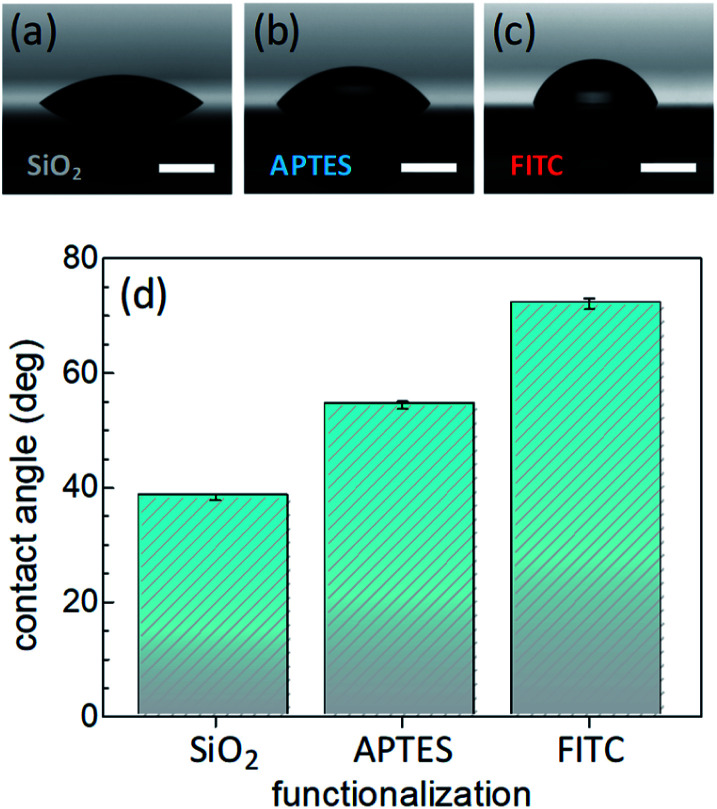
Pictures of a distilled water drop laid onto: (a) a silica layer on silicon substrate (Si@SiO_2_); (b) APTES grafted onto the silica layer (Si@SiO_2_@APTES); (c) FITC-functionalized system (Si@SiO_2_@APTES@FITC). Horizontal white bars correspond to 2 mm. (d) Histograms showing the evolution of the mean water contact angle value after each deposition step. Standard errors are indicated.

Surface wettability tests were performed by water CA measurements. Data revealed a decreasing surface wettability upon amino-modification by APTES and subsequent FITC linkage. In fact, the mean CA, which was found to be 38.3(0.5)° in the Si@SiO_2_ chip, increased up to 54.4(0.7)° after silanization with APTES^50^ and then up to 72.1(0.9)° upon functionalization with FITC^51^ (Fig. 2). These results could be easily rationalized as a decreased surface hydrophilicity due to the functionalization of the silica layer (bearing hydroxy groups, –OH) with the amino (–NH_2_) groups of APTES and then with FITC, which contains hydrophobic aromatic rings (Fig. 1b). Measurements carried out on different points of the sample surface confirmed the homogeneous deposition of the layers. This conclusion was supported at the nanoscale by AFM height images, showing lack of topographic reliefs and root mean square (RMS) values of the surface roughness lower than 1 nm (Fig. S1, ESI†).

**Fig. 3:**
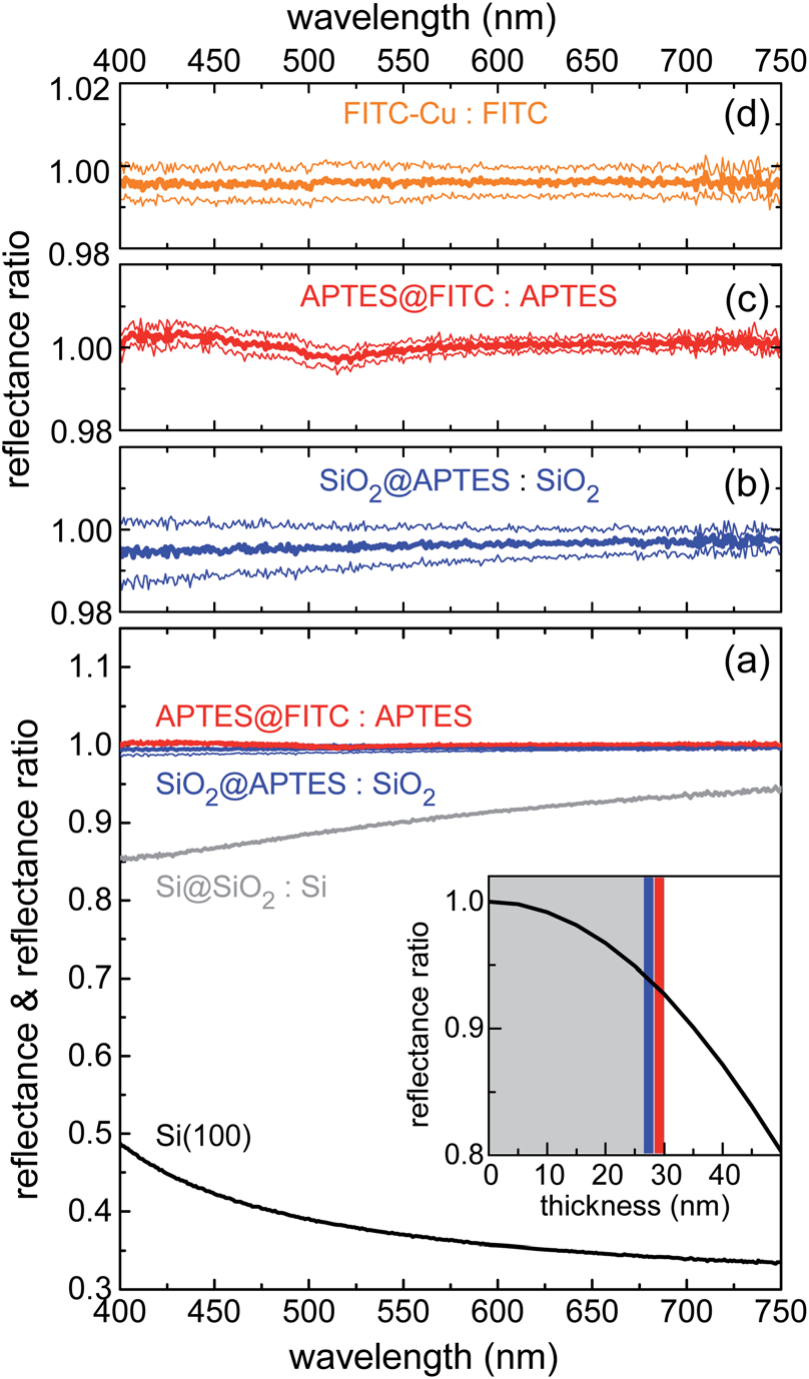
(a) Reflectance spectrum of n-doped (100)-oriented silicon substrate (black line); spectral reflectance ratios measured upon sequential layer deposition/functionalization: Si@SiO_2_-to-Si, Si@SiO_2_@APTES-to-Si@SiO_2_, and Si@SiO_2_@APTES@FITC-to-Si@SiO_2_@APTES ratios (grey, blue and red lines, respectively). Inset of panel (a): model estimate of Si@SiO_2_-to-Si reflectance ratio *vs.* SiO_2_ layer thickness (see text for details). Spectral reflectance ratio plotted on a vertical scale expanded around unity for: (b) Si@SiO_2_@APTES-to-Si@SiO_2_; (c) Si@SiO_2_@APTES@FITC-to-Si@SiO_2_@APTES; (d) Si@SiO_2_@APTES@FITC after dipping into a Cu^II^ aqueous solution (1.3 × 10^−4^ mol L^−1^). All panels: Thick lines are the mean signal values; thin lines are the mean values increased and decreased by their standard errors.

VIS reflectance spectroscopy was used to evaluate the thickness of the layers deposited through the various functionalization steps. Measurements were performed in hemispherical geometry to collect both specular and diffuse reflectance. Results are summarized in Fig. 3. Spectral reflectance ratio *R*_i_(*λ*)/*R*_i−1_(*λ*) was calculated after fabrication Step i. Silica deposition resulted in 10–15% decrease in reflectance across the visible spectrum (Fig. 3a), as the silica layer acted as an antireflection coating on silicon. Knowing the index of refraction of silica from previous work done with similar sol–gel precursor concentrations and physical deposition parameters,^52^ it was possible to calculate the thickness of the silica layer with high degree of accuracy. The estimated value of 28(1) nm implies that reflectance was very sensitive to any increase in thickness caused by subsequent functionalization steps. In the actual experimental setup, this sensitivity resulted to be ∼1 nm. In the inset of Fig. 3a, the reflectance ratio *R*/*R*_0_, where *R* (*R*_0_) is the reflectance of the Si@SiO_2_ structure (bare silicon chip) at 700 nm, is plotted against the SiO_2_ layer thickness.

The high sensitivity of the ratiometric reflectance to the thickness of the APTES and FITC layers was readily deduced from the slope of the black line at the intersection with the blue and red vertical bars, depicting the APTES and FITC layers, respectively. Fig. 3b and c show that reflectance remained practically unchanged upon functionalization of APTES and FITC, allowing to conclude that these functionalization steps were well-controlled and involved the deposition of monolayers. Indeed, a very small spectral dip was observed in the reflectance ratio measured after FITC deposition (Fig. 3c). This feature was found to be compatible with the optical absorption of a dense monolayer of FITC in the visible.

**Fig. 4:**
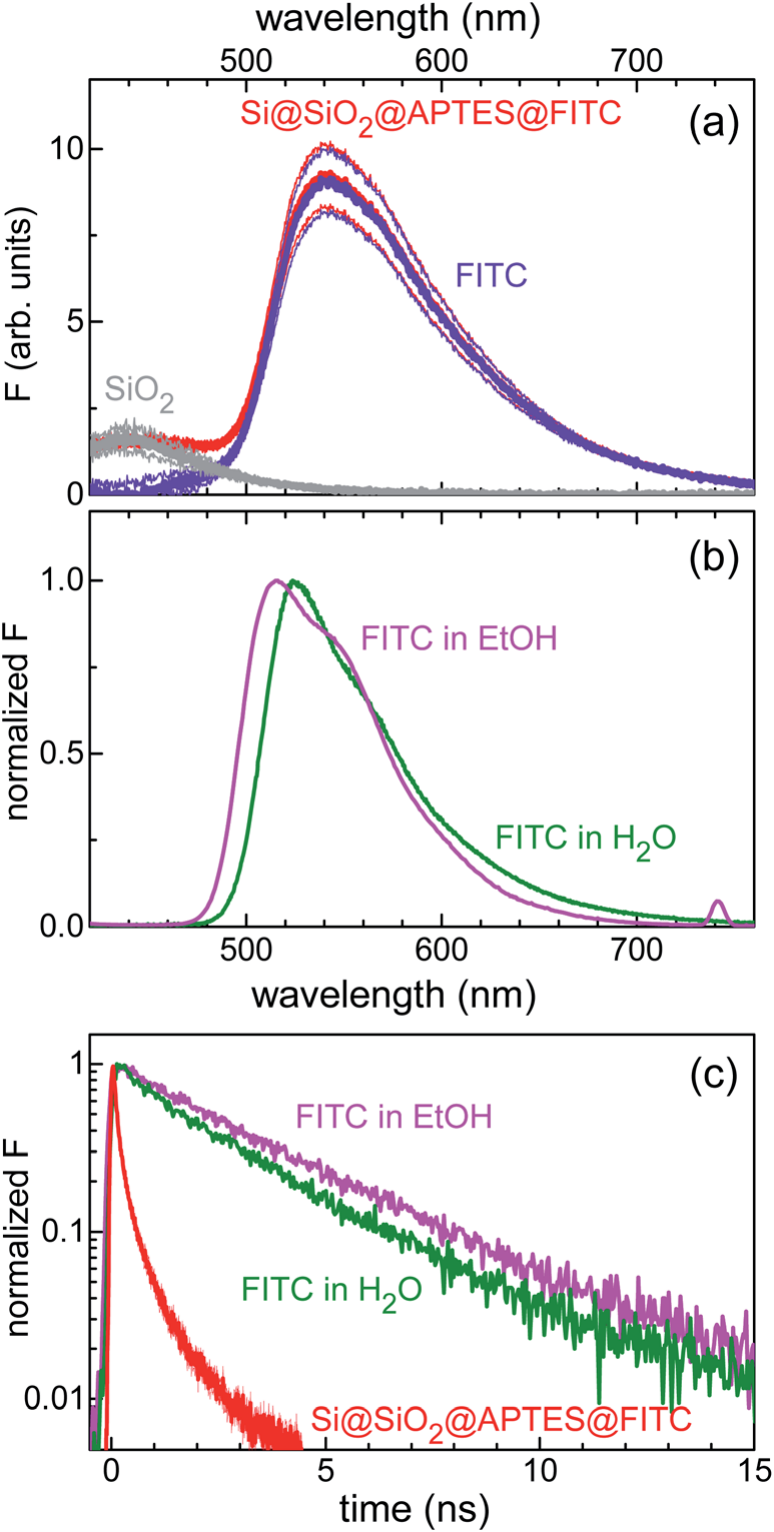
(a) Fluorescence intensity (F) spectrum of the Si@SiO_2_@APTES@FITC heterostructure. Red curves: fluorescence spectrum in air. FITC and SiO_2_ contributions to the whole spectrum are shown as the violet and grey lines, respectively. Thick lines are the mean signal values; thin lines are the mean values increased and decreased by their standard errors. (b) Normalized fluorescence spectrum of FITC in ethanol (1.2 × 10^−5^ mol L^−1^) and water (<10^−5^ mol L^−1^) solutions (magenta and olive line, respectively). (c) Normalized fluorescence decay curves of FITC in ethanol and water solution (magenta and olive line, respectively). Red curve: fluorescence decay transient of the device. Excitation wavelength was 355 nm.

Furthermore, combined continuous-wave/time-resolved fluorescence spectroscopy was applied to check for surface functionalization with FITC and gain information on the xanthene-centred fluorescence efficiency upon FITC linkage to the silicon surface. The fluorescence spectrum of the sensing device is shown in Fig. 4a. The main emission peak, centred at 540 nm (red curve), was readily assigned to the FITC xanthene unit, while comparative measurements on Si@SiO_2_ bare chip (grey curve) allowed for attributing the short-wavelength shoulder, centred at 420 nm, to the silica fluorescence spectrum. For reference, emission spectra were also collected for FITC dissolved in water and ethanol (Fig. 4b). FITC grafting resulted in loss of fluorescence efficiency, as deduced from the stretched exponential decay of the fluorescence intensity with reduced lifetime as compared to that of the nearly monoexponential decay observed in solution (Fig. 4c).

**Fig. 5:**
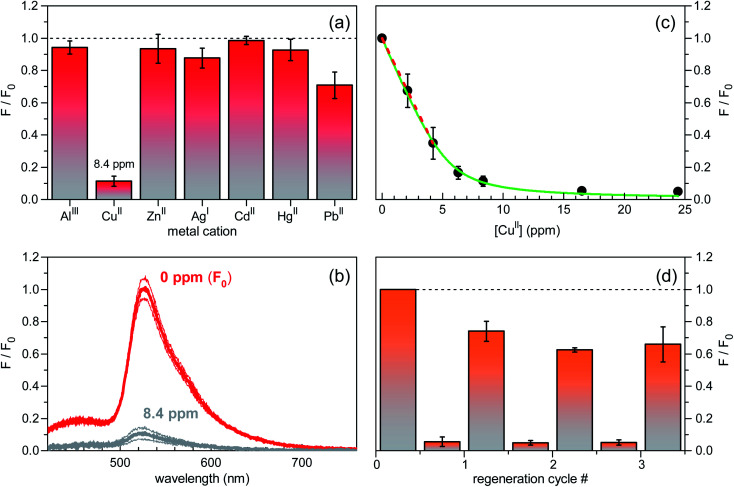
Sensing performance characterization of silicon-based fluorescent chips in aqueous MOPS buffer at pH = 7.2 (excitation wavelength of 355 nm). (a) Spectrally-integrated fluorescence intensity changes in response to equimolar (1.3 × 10^−4^ mol L^−1^) solutions of various metal ions. Cu^II^ mass concentration (in ppm) is also indicated. Standard error bars are reported. *F*_0_ is the reference value (at 0 ppm) of the fluorescence intensity signal. (b) Red lines: fluorescence spectrum of the unperturbed sensor; grey lines: fluorescence spectrum measured upon addition of 8.4 ppm of Cu^II^ ions. Thick lines are the mean signal values; thin lines are the mean values augmented and diminished by their standard errors. (c) Titration curve for the fluorescence intensity reduction (*F*/*F*_0_) *vs.* Cu^II^ concentration. Best fit of a linear decay function to the data in the low concentration regime is shown as dashed red line. A signal loss coefficient of 0.15 ppm^−1^ was inferred. Green line: best fit of model function, based on FITC–Cu^II^ interaction mechanism at equilibrium, to experimental data (see, also, Fig. S4, ESI†). (d) Sensor regeneration tests. Fluorescence turn-off behaviour of the device in a Cu^II^ water solution (8.4 ppm) and signal recovery after each regeneration cycle in EDTA solution. Here, *F*_0_ refers to the initial fluorescence intensity (at 0 ppm) before the regeneration test started.

A preliminary screening of the sensing performances was carried out on several metal cations (Al^III^, Cu^II^, Zn^II^, Ag^I^, Cd^II^, Hg^II^, Pb^II^) in MOPS buffer solutions at pH = 7.2, at a fixed molar concentration of 1.3 × 10^−4^ mol L^−1^ (Fig. 5a). Remarkably, among the investigated ions, only Cu^II^ resulted in a neat fluorescence turn-off behaviour (see also the fluorescence spectra in Fig. 5b) with a significant response on a short time scale of the order of tens of seconds. Sensitivity toward Cu^II^ was determined by fluorescence-intensity titration curves as a function of Cu^II^ concentration. In the low concentration regime below 5 ppm, the decrease in fluorescence intensity measured for increasing concentration was fitted with a linear decay function, yielding a signal loss coefficient of 0.15 ppm^−1^ or, equivalently, 15% reduction at 1 ppm level (Fig. 5c). Stabilization of fluorescence quenching was observed for Cu^II^ concentration values larger than 15 ppm. Finally, regeneration tests carried out in EDTA water solution showed stabilization of the fluorescence signal recovery level at ∼60% already after the second regeneration cycle (Fig. 5d), thereby demonstrating good reversibility of the surface–analyte interaction.

Several Cu^II^ sensing mechanisms could in principle be envisaged for the present hybrid platforms. Fluorescence sensing of Cu^II^ by a rhodamine B derivative linked to silica nanoparticles through a thiourea group was previously shown to yield Cu^II^ complexes exhibiting an optical absorption band partly overlapping with the fluorophore emission band, thus acting as fluorescence quencher *via* photoinduced Forster's resonance energy transfer (FRET).^18^ However, no additional absorption bands were detected by ratiometric reflectance after device dipping into a Cu^II^ aqueous solution (Fig. 3d).

Redox processes involving Cu^II^ reduction to Cu^I^, thiourea oxidation to a disulfide moiety and concomitant formation of Cu^I^–thiourea complexes were also demonstrated experimentally in solution.^53–55^ In the present fluorescent platforms, however, surface oxidation with formation of disulfide (–S–S–) bonds would presumably cause irreversible modification of the surface, in contrast with regeneration tests by using EDTA. Thiourea-induced copper(ii) reduction was therefore discarded as a possible metal-ion sensing mechanism.

Thiourea groups may indeed not be directly involved in Cu^II^ sensing. Experiments performed on FITC dilute solutions in ethanol/water, where thiourea groups are not present (Fig. S2, ESI†), showed that Cu^II^ can selectively interact with FITC, leading to a decrease in both absorbance and fluorescence intensity without formation of new optical absorption bands. FRET-type processes were therefore ruled out. A new FITC–Cu^II^ interaction mechanism was in turn suggested, which involves the formation of a fluorescein lactonic species where π-conjugation interruption leads to vanishing of the xanthene-centred optical transition moment^56^ and, hence, to fluorescence quenching. This mechanism (Scheme S2, ESI†) was further supported by mass and tandem mass spectrometry data (Fig. S3, ESI†). A model-based analysis of the fluorescence response for varying Cu^II^ concentration is also reported (Fig. S4, ESI†).

It is worth discussing on the limit of detection (LoD) of the reported fluorescent chips. LoD is defined as the analyte concentration that produces a response signal equal to a given threshold level, which is usually set to three standard deviations (*σ*) of the signal (*F*/*F*_0_) from its mean value. Although step-by-step characterization confirmed a good quality of the planar platforms, surface disorder appeared to result in non-negligible sample-to-sample fluctuations of the fluorescence intensity (Fig. 5b), whereas statistical noise and readout noise of the photodetection apparatus were found to be negligible. The resulting standard error bars of data points in Fig. 5c (3*σ* = 0.63 at [Cu^II^] = 2.1 ppm) currently set the Cu^II^ LoD to 4.1 ppm, a value that is close to current limits for copper in drinking waters. The large sensitivity value of 0.15 ppm^−1^ could be exploited to greatly improve the LoD of the fluorescent silicon platforms upon reducing chip-to-chip signal fluctuations.^18^

Another point of interest is represented by the fluorescence efficiency that can be achieved upon fluorophore linkage to the silicon substrate. The strong reduction reported for the FITC fluorescence lifetime upon surface grafting (180 ps against the 2.6 ns value found in water solution, Fig. 4c) hints to a strong sensitivity of FITC fluorescence to surface loading, possibly arising from the sizable absorption-emission spectral overlap, which causes fluorescence self-quenching through FITC-to-FITC photoinduced energy transfer.^37^ This issue, often encountered in bioanalytical applications of fluorescence, opens up to the use of organic dyes with shorter critical (Foerster's) energy transfer distance and, hence, less sensitive to fluorescence self-quenching.

## Conclusions

Silicon-based fluorescent chips for the detection of metal ions in water were reported, where the xanthene-based fluorophore, FITC, was covalently linked to an amino-silanized silicon surface *via* thiourea formation. These solid-state hybrid platforms exhibited selectivity towards copper(ii) with good detection limit, competitive sensitivity, and regeneration capability using a metal–chelating agent. An original FITC–Cu^II^ reaction mechanism involving the formation of a lactonic fluorescein species, exhibiting disappearance of the fundamental optical transition moment, is proposed as a possible cause of fluorescence quenching. The sensing platform architecture, where recognition/fluorescent units are integrated on a silicon chip *via* a layer-by-layer functionalization approach, is very versatile owing to the possibility of tuning the selectivity to other metal ions or different types of analytes by changing the fluorophore or the anchoring group. This represents a viable strategy towards silicon-integrated fluorescent devices for remote detection of a range of metal ions in water. In the present solid-state planar platforms with high dye loading, spatial uniformity and quantum efficiency of the fluorophore are important issues that require further investigations before determining matrix effects, such as the role of pH and interfering ions, in real water samples.

## Experimental section

### Materials

Tetraethoxysilane (TEOS, purity >99%), aminopropyltriethoxysilane (APTES, purity >97%), fluorescein isothiocyanate (FITC, purity >95%), 3-(*N*-morpholino)propanesulfonic acid (MOPS, purity >99%), ethylenediaminetetraacetic acid (EDTA, purity >99%), metal perchlorates (Al^III^, Cu^II^, Zn^II^, Ag^I^, Cd^II^, Hg^II^, Pb^II^, purity >99%), absolute ethanol (EtOH, purity >99%), and hexane (purity >99%), were used as received without further purification. All reactants and solvents were purchased from Sigma Aldrich, exception made for FITC (Tokyo Chemical Industry, TCI). Silicon wafers (100 orientation, P/B doped, resistivity 5–10 Ω cm, thickness 380 μm) were purchased from Siegert Wafer.

### Silica synthesis and deposition

The silica sol precursor was prepared by mixing TEOS, EtOH and distilled water under stirring at room temperature (RT). Subsequently, HCl was added to the sol and the mixture was maintained under stirring at 60 °C overnight. The molar ratio of the starting solution was TEOS : H_2_O : EtOH : HCl = 1 : 5 : 6 : 0.065. A diluted solution was prepared by mixing freshly prepared TEOS solutions with a proper amount of EtOH (volume ratio 1 : 10) in a closed vessel at RT. Silica films were deposited on 100-oriented silicon substrates cut into ∼15 × 15 mm^2^ pieces for morphological and optical characterization, and ∼7 × 7 mm^2^ pieces for sensing measurements. Prior to coating, the silicon substrates were cleaned with water soap (in ultrasonic bath at 50 °C for 15 min), distilled water, acetone, and finally rinsed with isopropanol and dried in a nitrogen flow. Ultrathin (∼30 nm) silica films were obtained by spin coating at 7000 rpm for 30 s. After deposition, samples were dried at RT for 24 h.

### Silica@APTES functionalization

The functionalization of the ultrathin silica films on silicon substrates with amino groups was done by immersion of the substrates in a 1 mM APTES/hexane solution for 10 s. The films were then rinsed with hexane followed by acetone to remove the excess of APTES.

### Silica@APTES@FITC functionalization

A FITC/EtOH mixture was prepared by dissolving 8 mg of FITC in 10 mL of EtOH. Silica@APTES films on silicon substrates, *vide supra*, were then immersed in the FITC solution for 30 min. The reaction was performed at RT in dark conditions. Upon completion of the reaction, the FITC-functionalized silicon substrates were rinsed with EtOH and acetone to remove the excess of FITC, and then kept in 20 mL of milli-Q water for 20 min before characterization measurements.

### Morphological characterization

A NT-MDT Solver-Pro atomic-force microscopy (AFM) instrument was used to study the topography of the sample surface. AFM measurements were performed at 0.5–1 Hz scan speed in semicontact mode in air. Topography image analysis and calculation of surface roughness were performed by using WSxM 5.0 Develop3.2 software.^57^ The measurements were performed on at least three different points of the same sample to assess the uniformity of the layers.

### Surface wettability experiments

Water contact angle (CA) measurements were performed at 22 °C by using a Kruss drop shape analyzer (DSA 30S) and analyzed by the Kruss Advance software. A sessile drop method was used to measure the contact angle of a 1 μL distilled water drop. The measured CA was the average between the left and right contact angles. The measurements were performed on at least three different points of the same sample to assess the uniformity of the layers.

### Optical spectroscopy and fluorescence lifetime measurements

Reflectance spectroscopy measurements were performed under sample direct illumination in a dual-beam spectrophotometer (Agilent Technologies Cary 5000 UV-Vis-NIR) equipped with a diffuse reflectance accessory. Fluorescence spectra for samples in air and in aqueous 3-(*N*-morpholino)propane sulfonic acid (MOPS) buffer in a quartz cuvette were measured under 355 nm irradiation of the samples by a passively Q-switched powerchip laser (Teem Photonics PNV-M02510) operating in pulsed regime (350 ps pulses, 1 kHz repetition rate). The fluorescence was spectrally resolved using a single-grating spectrometer (Princeton Instruments Acton SpectraPro 2300i) and acquired by a thermoelectrically cooled Vis CCD camera (AndorNewton^EM^). Fluorescence lifetime were measured in air under sample irradiation at 350 nm by an optical parametric amplifier (Light Conversion TOPAS-C) pumped by a regenerative Ti:Sapphire amplifier (Coherent Libra-HE), delivering 200 fs-long pulses at 1 kHz repetition rate. The fluorescence was spectrally dispersed using a single-grating spectrometer (Princeton Instruments Acton SpectraPro2300i) and acquired by a Vis streak camera (Hamamatsu C1091). Pump fluence was kept below 50 μJ cm^−2^ per pulse in all fluorescence experiments to prevent sample degradation.

### Sensing measurements

Sensing performances towards different metal cations were assessed by fluorescence spectroscopy with the silicon-chip sensors placed in a 1 cm-thick quartz cuvette. The cuvette was then filled with 1500 μL of aqueous MOPS buffer (70 mM, pH = 7.2) using fresh distilled water purified by a Milli-Q system (Millipore), and 60 μL of 0.1 mM water solution of various perchlorate salts (Al^III^, Cu^II^, Zn^II^, Ag^I^, Cd^II^, Hg^II^, Pb^II^) was added. Sensitivity towards Cu^II^ ions was evaluated through fluorescence titration experiments in the 0–120 μL range. A waiting time of 3 min was set before each measurement to ensure that the surface–analyte interaction had reached equilibrium. Surface regeneration after sensing tests was accomplished by sample sonication in 3 mL of EDTA solution (0.1 M) for 10 min. Three generation cycles were performed on each sample.

### Mass spectrometry

Mass spectra were recorded using a triple quadrupole QqQ Varian 310 MS mass spectrometer using the atmospheric-pressure Electrospray Ionization (ESI) technique. FITC solutions (20 μL) were injected into the ESI source by a Rheodyne® model 7125 injector connected to a HPLC Varian 212 LC pump, with a 50 μL min^−1^ methanol flow. Experimental conditions: Dwell time 2 s, needle voltage 3000 V, shield voltage 600 V, source temperature 60 °C, drying gas pressure 20 psi, nebulizing gas pressure 20 psi, detector voltage 1600 V. Mass spectra were recorded in the 100–600 *m*/*z* range. Collision-Induced Dissociation (CID) tandem mass (MS/MS) experiments were performed using argon as the collision gas (1.8 psi). Collision energy was varied from 20 to 40 eV.

## Author contributions

M. L. M. and F. Q. designed and directed the research project. M. O. and C. F. fabricated the sensing devices by making all silicon functionalization steps. M. O., C. F., D. M., E. C. and F. Q. performed the experiments and analyzed the data. M. O., C. F., E. C., M. L. M. and F. Q. wrote the manuscript. All authors contributed to the final interpretation of the experimental results and critically revised the manuscript. All authors have read and approved the final version of the manuscript.

## Conflicts of interest

There are no conflicts to declare.

## Supplementary Material

RA-011-D1RA02695J-s001
