# Supplementary material for: Silicon-based fluorescent platforms for copper(ii) detection in water
Source: RSC Adv. 2021 Apr 26;11(26):15557–64. doi: 10.1039/d1ra02695j (PMC9029085; doi:10.1039/d1ra02695j)
Supplement: RA-011-D1RA02695J-s001 [file RA-011-D1RA02695J-s001.pdf]

Electronic Supplementary Material (ESI) for RSC Advances.
This journal is © The Royal Society of Chemistry 2021

Electronic Supplementary Information for

Silicon-based Fluorescent Platforms for Copper(II) Detection in Water

Mariangela Oggianu,[a,c] Cristiana Figus,[b] Suchithra Ashoka-Sahadevan,[a,c] Noemi Monni,[a,c] Daniela Marongiu,[b] Michele Saba,[b] Andrea Mura,[b,c] Giovanni Bongiovanni,[b] Claudia Caltagirone,[a,c] Vito Lippolis,[a,c] Carla Cannas,[a,c] Enzo Cadoni,[a,c] Maria Laura Mercuri *[a,c] and Francesco Quochi *[b,c]

[a] *Dipartimento di Scienze Chimiche e Geologiche, Università degli Studi di Cagliari, Complesso Universitario di Monserrato, I-09042 Monserrato (CA), Italy*

[b] *Dipartimento di Fisica, Università degli Studi di Cagliari, Complesso Universitario di Monserrato, I-09042 Monserrato (CA), Italy*

[c] *INSTM, Cagliari Unit, Via Giuseppe Giusti, 9, I-50121 Firenze, Italy*

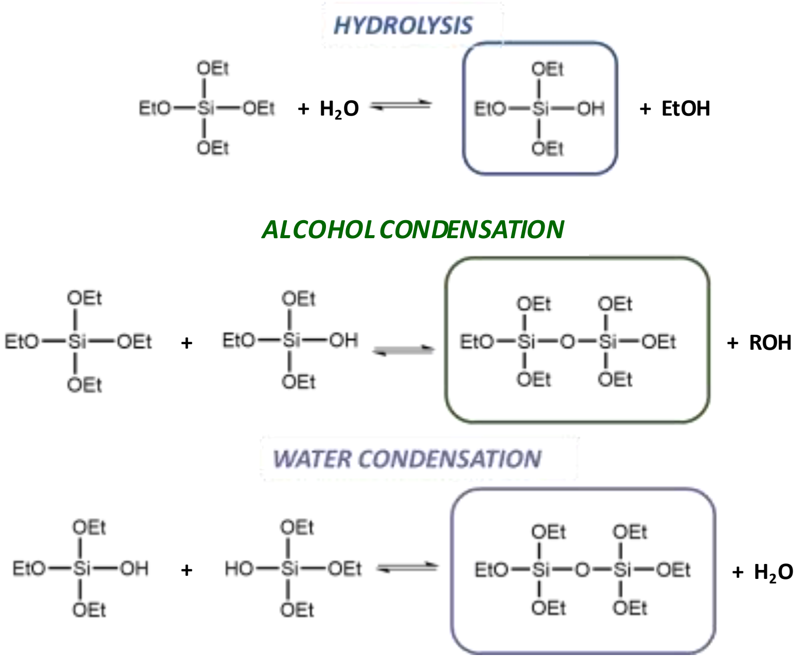

Scheme S1 Reactions involved in the sol-gel process with tetraethoxysilane as the silica precursor.

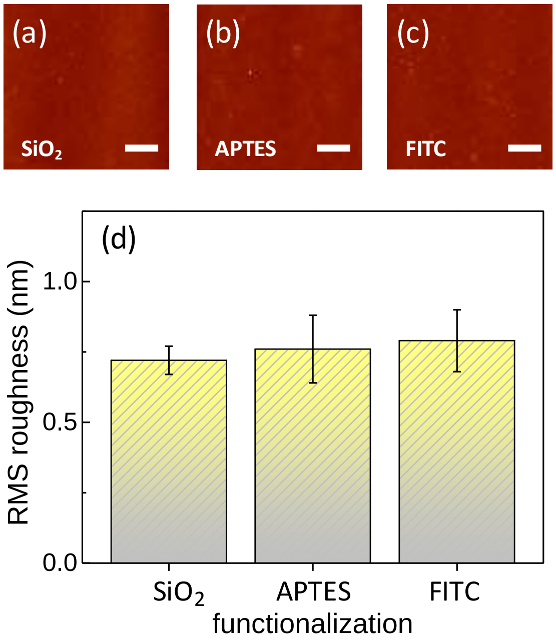

Fig. S1 AFM surface topography of silicon-based heterostructures. (a) Image of the surface of a sol-gel silica layer (Si(100)@SiO_2 heterostructure). (b) Same as (a) but for an Si(100)@SiO_2@APTES heterostructure. (c) Same as (b) but for a Si(100)@SiO_2@APTES@FITC heterostructure. White bars: 10 μm. (d) Histogram depicting the evolution of the RMS surface roughness after each deposition step. Standard errors are indicated.

FITC-BASED Cu^{II} SENSING MECHANISM

Mechanism description. An intramolecular charge-transfer process, triggered by Cu^{II} coordination to the quinonic oxygen atom at the 3 position of the xanthene moiety, is envisaged as a possible cause of the observed fluorescence quenching of the Si@SiO$_2$@APTES@FITC device (Scheme S2). The process leads to the formation of a spiranic lactone, resulting in fluorescein conjugation interruption, with consequent vanishing of the fundamental optical transition moment and fluorescence quenching.[1] This process, which is alternative to Cu^{II}-S interaction at the thioureidic groups of the device, clearly distinguishes the Si@SiO$_2$@APTES@FITC platform from thiourea-based fluorescent chemosensors reported in literature.

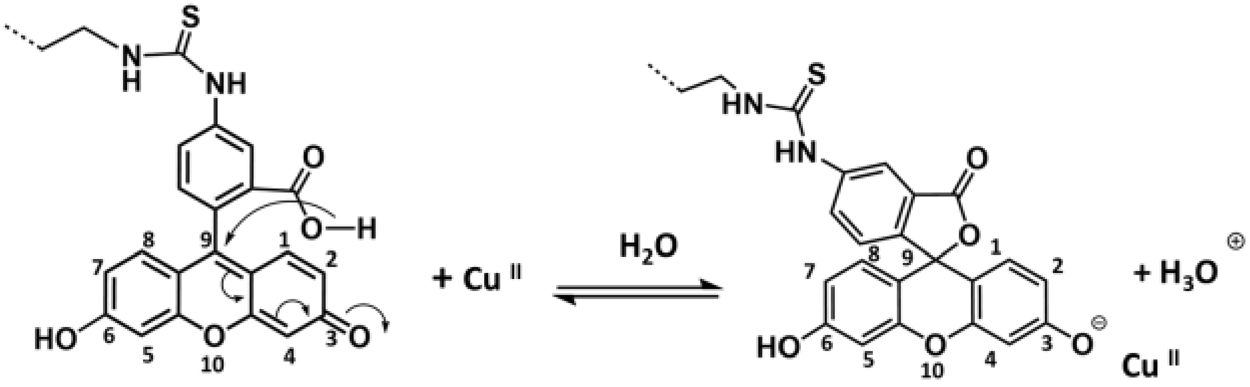

Scheme S2 Cu^{II}-triggered charge-transfer to the quinonic oxygen and lactonic fluorescein formation at the surface of the Si@SiO$_2$@APTES@FITC platform (Si@SiO$_2$ substrate not shown).

Control measurements in solution. In order to provide experimental support to the suggested FITC-Cu^{II} interaction mechanism, FITC, FITC/Zn^{II} and FITC/Cu^{II} dilute solutions in 1:1 v/v EtOH/H_2O and pure EtOH were studied by Fluorescence, UV-Vis-Spectroscopy, and ESI mass (MS) and Collision-Induced Dissociation (CID) tandem mass (MS/MS) Spectrometry.

Addition of Cu^{II} to a FITC solution led to reduction in both Vis absorbance and fluorescence intensity of FITC (Fig. S2). In EtOH/H_2O, reduction in fluorescence intensity was of the same order of magnitude as that reported on the Si@SiO_2@APTES@FITC sensor (Fig. S2a), whereas, in pure EtOH, it resulted in a 10-fold increase in response towards Cu^{II} as compared to the on-chip device (Fig. S2b). The same experiment performed on FITC/Zn^{II} solutions produced no response in EtOH/H_2O, hence demonstrating FITC selectivity towards Cu^{II} in aqueous environment; in EtOH, a large signal reduction in both absorbance and fluorescence intensity was instead observed, showing a significant response also to Zn^{II}. Correlated reductions in Vis absorbance and fluorescence intensity, that is, the progressive conversion of FITC into a colorless and nonfluorescent species, is consistent with the formation of spiranic lactones in the FITC/metal-ion mixed solutions.

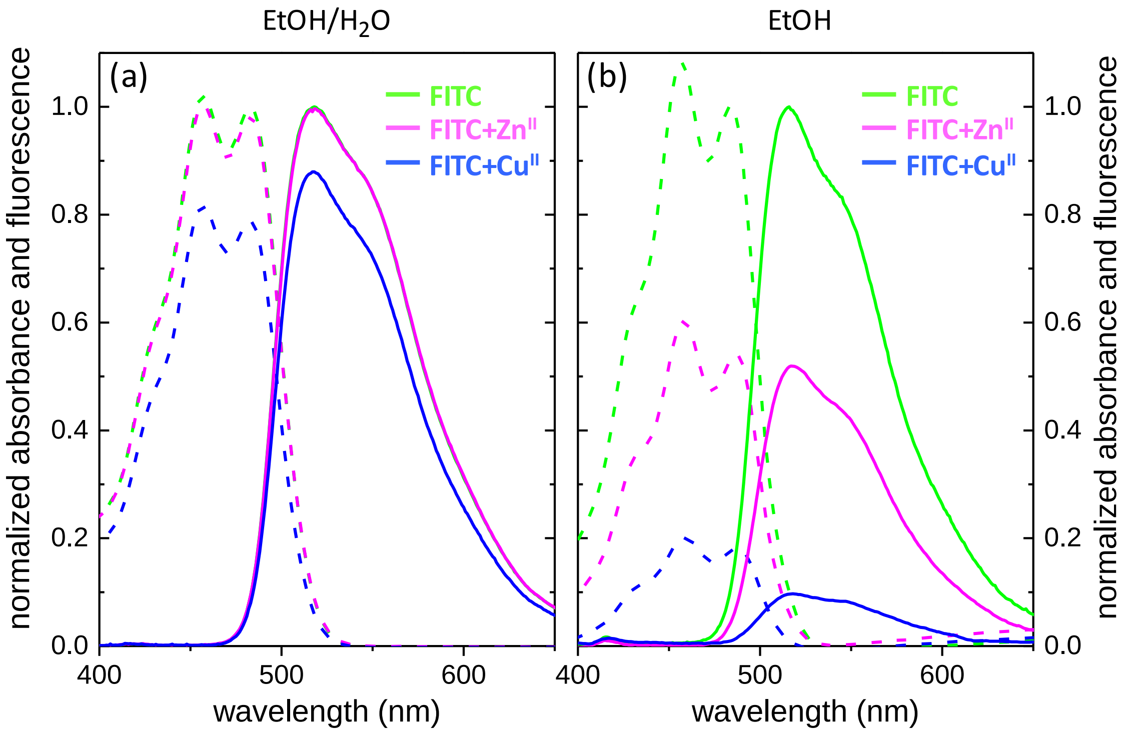

Fig. S2 Absorbance spectra (dashed lines) and fluorescence spectra, photoexcited at 370 nm (solid lines) of FITC (green), FITC/Zn^{II} (magenta) and FITC/Cu^{II} (blue) in (a) 1:1 v/v EtOH/H_2O, and (a) EtOH. FITC concentration is 1.2×10^{-5} mol L^{-1}; Zn^{II} and Cu^{II} concentrations are 1.2×10^{-4} mol L^{-1} in panel (a) and 1.2×10^{-5} mol L^{-1} in panel (b). Data are normalized to the 483 nm absorbance peak value and maximum fluorescence intensity of pure FITC solutions.

ESI-MS spectra of FITC and equimolar FITC/Cu^{II} ethanol solutions, acquired in positive ion mode, are reported in Fig. S3a, showing no direct evidence for FITC-Cu^{II} adducts.

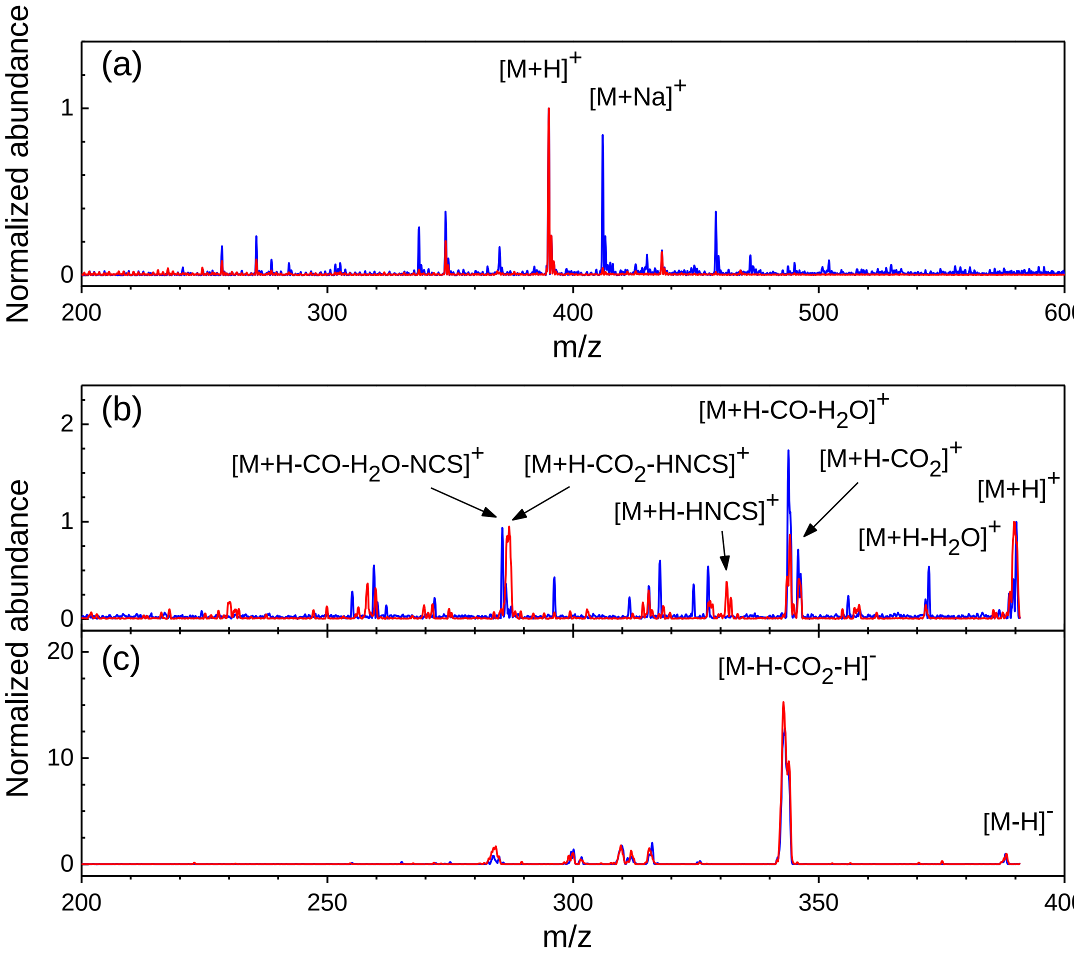

Fig. S3 ESI-MS spectra in positive ion mode of FITC (a), ESI-CID MS/MS spectra in positive mode (b), and ESI-CID MS/MS spectra in negative mode (c) of FITC (blue lines) and equimolar FITC/Cu^{II} solution (red lines). All spectra are normalized to the $[M\pm H]^{\pm}$ parent peak intensity. Relevant peak assignments are displayed.

In positive ion mode, the lactonic anion is supposed to be stabilized in a double protonation process, shown in Scheme S3.

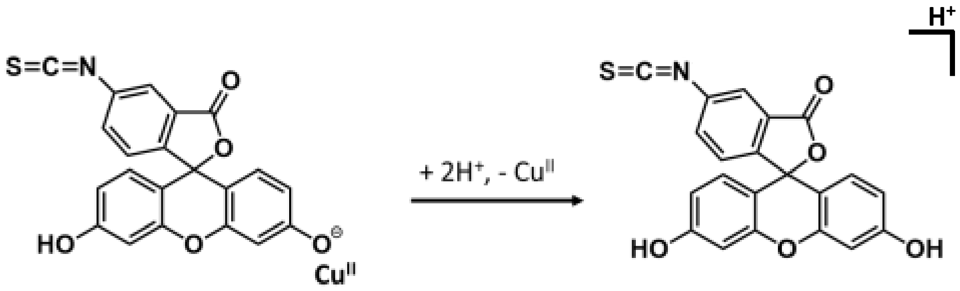

Scheme S3 Double protonation process of lactonic fluorescein anion by ESI in positive ion mode.

Structural isomers obtained in positive ion mode could be distinguished in ESI-CID MS/MS spectra. $[M+H]^+$ fragmentation spectrum of the pure FITC sample revealed a much larger loss of water, carbon monoxide and carbon dioxide than that of FITC/Cu^{II} solution, which is consistent with the formation of a lactonic species in the FITC/Cu^{II} solution; more in detail, $[M+H-H_2O]^+/[M+H]^+$ ≅0.55 for the FITC solution and ≅0.15 for the FITC/Cu^{II} solution (Fig. S3b). Further confirmation of the formation of lactonic fluorescein comes from the observation of the $[M+H-HNCS]^+$ mass peak (m/z = 331), only in the FITC/Cu^{II} solution. The fragmentation mechanism is shown in Scheme S4.

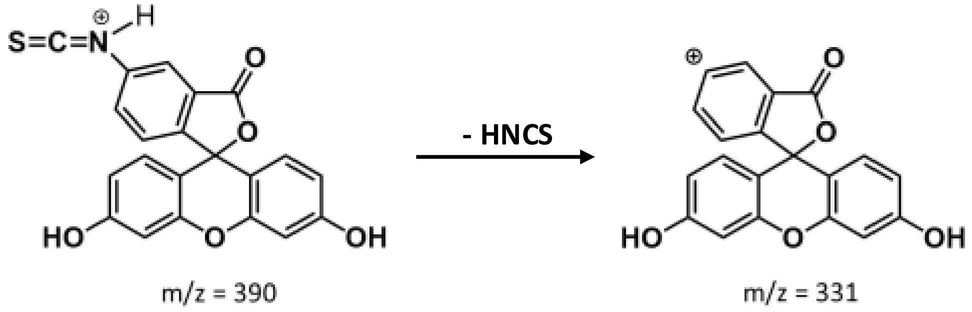

Scheme S4 Fragmentation of protonated lactonic fluorescein leading to the formation of phenyl carbocation with moderate stability.

Remarkably, in protonated open FITC, the same fragmentation mechanism would result in excess of positive charge on the phenyl ring, compared to the lactonic form, owing to the negative mesomeric effect by the quinonic oxygen *via* the phenyl/xanthene conjugated system (not shown), and can thus be ruled out.

In negative ion mode, FITC lactonic form is transformed into the open structure (Scheme S5), and, consequently, FITC fragmentation spectrum should be the same in both FITC and FITC/Cu^{II} solutions.

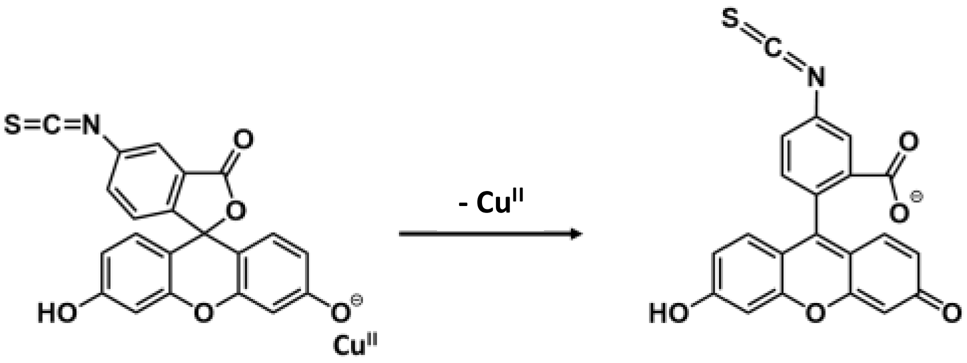

Scheme S5 Lactonic-into-open fluorescein anion conversion by ESI in negative ion mode.

This was actually verified experimentally: both FITC and FITC/Cu^{II} negative ion ESI-CID (20 eV) MS/MS spectra (Fig. S3c) exhibited a very intense base peak at m/z = 343, which was identified as $[M\text{-}H\text{-}CO_2\text{-}H]^{-}$, that is, a stable radical anion obtained from deprotonated FITC, through sequential loss of CO_2 and a hydrogen atom (Scheme S6).

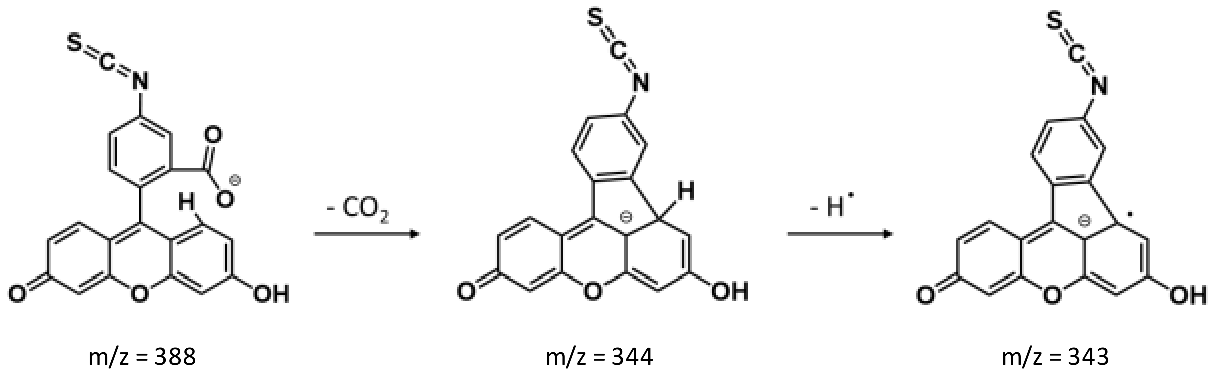

Scheme S6 Deprotonated FITC undergoing sequential loss of carbon dioxide and a hydrogen atom towards the formation of a cyclic radical anion with m/z = 343.

Results of ESI-mass and CID MS/MS experiments on ethanol solutions were confirmed in EtOH/H_2O solutions.

Overall, optical and ESI-CID MS/MS data are consistent with the formation of a lactonic fluorescein species in the FITC/Cu^{II} solution, suggesting the possibility of a direct FITC-Cu^{II} interaction mechanism at the surface of the Si@SiO_2@APTES@FITC heterostructure.

MODEL-BASED ANALYSIS OF Cu^{II} SENSING

The proposed FITC-Cu^{II} reaction mechanism was used to model the response of the Si@SiO_2@APTES@FITC platform vs. Cu^{II} concentration. To this aim, chemical equilibrium was considered in an aqueous buffer solution (at pH = 7.2) as follows:

$$\frac{[\mathrm{LACT^+}]_{\mathrm{eq}} \cdot [\mathrm{H_3O^+}]_{\mathrm{eq}}}{\left([\mathrm{FITC}] - [\mathrm{LACT^+}]_{\mathrm{eq}}\right) \cdot \left([\mathrm{Cu^{II}}] - [\mathrm{LACT^+}]_{\mathrm{eq}}\right)} = K_{\mathrm{eq}}\,, \tag{S1}$$

where $[\mathrm{LACT^+}]_{\mathrm{eq}}$ is the equilibrium concentration of lactonic fluorescein–Cu^{II} adducts, $[\mathrm{H_3O^+}]_{\mathrm{eq}} = 10^{-\mathrm{pH}}$, $[\mathrm{FITC}]$ and $[\mathrm{Cu^{II}}]$ are the FITC and Cu^{II} initial concentrations, respectively, and K_{eq} is the equilibrium constant. After solving Eq. S1 for $[\mathrm{LACT^+}]_{\mathrm{eq}}$, knowing that the

lactonic species is nonfluorescent, fluorescence intensity dependence on Cu^{II} concentration was calculated as

$$\frac{F}{F_0} = 1 - \frac{[\mathrm{LACT}^+]_{\mathrm{eq}}}{[\mathrm{FITC}]}, \tag{S2}$$

where F is the fluorescence intensity and F_0 is the reference fluorescence intensity for $[\mathrm{Cu^{II}}] = 0$. The expression for F/F_0 in Eq. S2 was fitted to the experimental fluorescence titration data with $[\mathrm{FITC}]$ and K_{eq} as free parameters. The result is shown in Fig. S4 and Fig. 5c. Best fit yielded $[\mathrm{FITC}] = 8.4 \times 10^{-5}$ mol L^{-1} and $K_{\mathrm{eq}} = 9.8 \times 10^{-3}$.

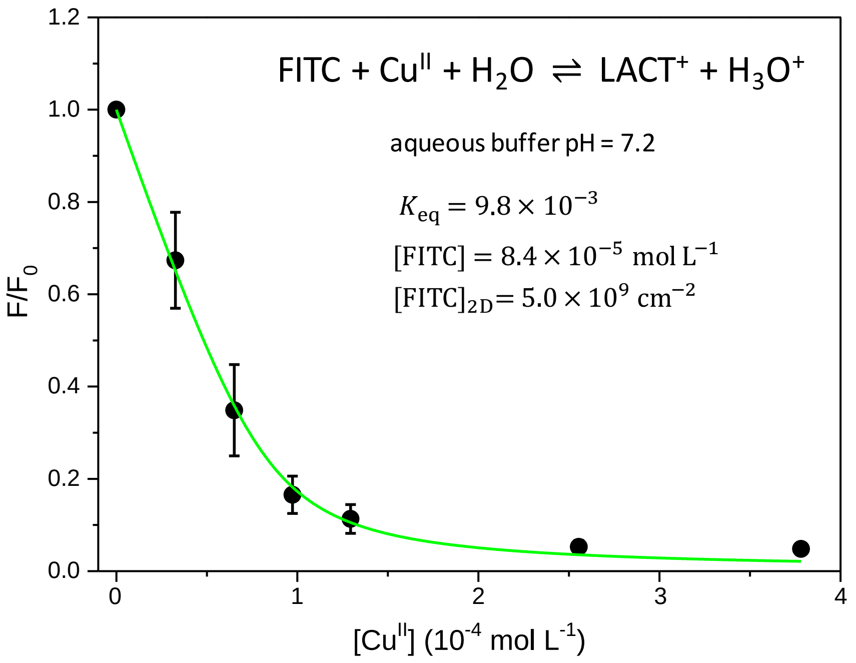

Fig. S4 Black points: Normalized fluorescence of Si@SiO_2@APTES@FITC sensor vs. Cu^{II} concentration. Green line: Best fit of model function, based on FITC-Cu^{II} interaction mechanism at equilibrium, to experimental data (see text for details).

$[\mathrm{FITC}]$ actually refers to a three-dimensional situation with the fluorophores in solution. The equivalent two-dimensional value applying to the on-chip sensor, $[\mathrm{FITC}]_{\mathrm{2D}}$, was estimated as

$$[\mathrm{FITC}]_{\mathrm{2D}}\,(\mathrm{cm^{-2}}) = [\mathrm{FITC}](\mathrm{mol\,L^{-1}}) \cdot \frac{N_{\mathrm{A}}}{1000}(\mathrm{L\,mol^{-1}cm^{-3}}) \cdot h(\mathrm{cm})\,, \tag{S3}$$

where N_{A} is Avogadro's number and h is the depth of the FITC layer. For $h = 1$ nm, one obtains $[\mathrm{FITC}]_{\mathrm{2D}} = 5.0 \times 10^9\ \mathrm{cm^{-2}}$, a reasonable value for fluorophores grafted on a silanized silicon substrate.

REFERENCES

1 C. Liu, S. Huang, H. Yao, S. He, Y. Lu, L. Zhao and X. Zeng, *RSC Adv.*, 2014, **4**, 16109–16114.
